# Biosocial Determinants of Health Among Patients with Chronic Liver Disease and Liver Cancer

**DOI:** 10.3390/cancers17050844

**Published:** 2025-02-28

**Authors:** Tagari Samanta, Jun Hyoung Park, Benny Abraham Kaipparettu

**Affiliations:** 1 Department of Molecular and Human Genetics, Baylor College of Medicine, Houston, TX 77030, USA; tagari.samanta@bcm.edu (T.S.); junhyoup@bcm.edu (J.H.P.); 2 Dan L. Duncan Comprehensive Cancer Center, Baylor College of Medicine, Houston, TX 77030, USA

**Keywords:** All of Us research program, Hispanic, African American, chronic liver disease, liver cancer, socioeconomic status

## Abstract

Background: Metabolic disorders and chronic liver disease (CLD) play crucial roles in the development and progression of liver cancer (LC). Since the ethnic minority population increasingly suffers from CLD and LC, it is vital to understand the biosocial factors contributing to CLD and LC. The ’All of Us’ database, with significant participation from minority populations, provides a valuable tool for studies in different racial/ethnic groups. Using different databases, including the ‘All of Us’ and ‘The Cancer Genome Atlas’, this study aimed to understand the biosocial factors contributing to CLD and LC. Methods: Using ‘All of Us’ data, confounding factors like the lack of immunization, comorbidities, and socioeconomic status (SES) barriers were analyzed in a cohort of 33767 CLD [non-alcoholic fatty liver disease, alcoholic liver disease, and Hepatitis B and C] patients. Among the 556 LC patients in the ‘All of Us’ database, 92% had CLD. Since hypoxanthine is known to be increased in the urine of LC patients, purine metabolic pathway genes were analyzed using different databases and validated using publicly available RNASeq data. Results: We identified several confounding factors associated with CLD in Hispanic (HA) and African American (AA) populations compared to the non-Hispanic White (NHW) populations. HA and AA CLD patients suffer from high SES barriers. While most of the genes related to the purine metabolic pathway were upregulated in LC, xanthine dehydrogenase (XDH), which converts hypoxanthine to uric acid, showed a downregulation in the tumor compared to the normal tissues. The TCGA data among different racial/ethnic groups showed that only in Asian (AN) LC tumors the XDH expression was significantly lower compared to the NHW. The decreased XDH mRNA expression in AN LC compared to benign tissues was further validated using publicly available RNAseq datasets. Survival analysis confirmed poor overall survival among the AN LC patients with lower XDH expression in their tumors. Conclusions: Our study identified several confounding factors contributing to the minority CLD population. This study also identified decreased XDH expression as a critical metabolic alteration that has clinical significance in AN LC patients.

## Simple Summary

Liver cancer (LC) is one of the major consequences of chronic liver disease (CLD) because more than 90% of LC is developed from CLD. Racial and ethnic differences exist in the occurrence and biology of CLD and LC. Thus, we analyzed the social and biological factors contributing to racial/ethnic differences in the CLD and LC patients. Our study showed that Hispanic and African American CLD patients have a high social barrier compared to non-Hispanic White patients. Similarly, Asian LC patients show a metabolism-related defect in their tumors compared to other racial/ethnic groups. Overall, our data suggest that social and/or biological differences exist between ethnic minority patients with CLD and LC.

## 1. Introduction

Chronic liver disease (CLD) and liver cancer (LC), specifically hepatocellular carcinoma (HCC), pose an increasing health challenge in the USA, impacting millions of individuals and resulting in substantial morbidity and mortality [1]. Metabolic disorders and CLD play a crucial role in the development and progression of LC [2,3]. Racial/ethnic minority groups, such as Asian (AN), Hispanic (HA), and African American (AA), increasingly suffer from CLD and LC [4]. Thus, the impact of social and biological factors contributing to CLD and LC requires further investigation.

The number of new cases of LC per year is predicted to increase by 55.0% globally between 2020 and 2040, with a possible 1.4 million people diagnosed in 2040 [5]. More than 90% of LC cases arise in individuals with CLD [2,6,7]. CLD condition encompasses various liver disease subtypes, such as non-alcoholic fatty liver disease (NAFLD), alcoholic liver disease (ALD), Hepatitis B, and Hepatitis C [8]. According to the current data from the Centers for Disease Control and Prevention (CDC), CLD ranks as the ninth leading cause of death in the United States [9]. Advanced CLD can lead to liver cirrhosis and significantly elevate the risk of developing LC [10].

As per the 2022 American Community Survey (ACS) report from the United States CENSUS Bureau (www.census.gov, accessed on 4 September 2024) [11], the HA population is the second-largest ethnic group (19%) in the USA. AAs comprise 14%, and ANs comprise 7% in the USA. A previous study reported that Mexican Americans and Black Americans have a greater risk of developing liver disease than their White counterparts [12]. Moreover, ANs in the USA have higher rates of chronic hepatitis B and C, which contribute to higher rates of liver disease and LC compared to other groups [13,14]. New approaches like the ’biosocial determinant of health’ [15] analysis are necessary to describe molecular factors or physiological processes that reflect the complexities of health inequity and biological factors to shed light on the ongoing issue of CLD and LC disparities. The liver is the largest internal organ for controlling metabolism, and metabolic disruption is closely associated with LC [16]. Identifying critical metabolic changes during LC progression is crucial for understanding the etiology of LC and its therapeutic intervention.

However, understanding of the factors associated with racial disparity among the under-represented population is limited, mainly due to the lack of reliable databases with a considerable representation of the minority population. Fortunately, in recent efforts to improve the awareness of diseases among different populations, few databases are available with substantial data from minority populations. The ’All of Us’ database [17,18] offers a valuable platform for exploring the complex interplay between healthcare, behavioral, and social factors within the same population. Similarly, databases like ‘The Cancer Genome Atlas’ (TCGA) [19] and ‘Gene Expression database of Normal and Tumor tissues’ (GENT2) [20] provide a better opportunity to compare different cancer studies on a single platform. The overall objective of this study was to understand the ’biosocial determinant of health’ factors influencing the disparity of CLD subtypes and LC among the race/ethnic minority population.

## 2. Materials and Methods

### 2.1. The Population Data Used for This Study

*All of Us database*: The National Institutes of Health’s ‘All of Us’ Research Program has a large collection of data from various ethnic/racial groups living in the United States [17]. These data are a collection of various survey data from participants’ lifestyle, overall health, and healthcare access. In the electronic health record (EHR), the health conditions of the ‘All of Us’ database are classified according to ICD-9 and ICD-10 codes. All participants provided written informed consent to the ‘All of Us’ data program. Using ICD-9 and ICD-10 codes, we identified the LC (ICD-10 code: C22.0 and ICD-9 code: 155.0) and CLD cohorts enrolled in the ‘All of Us’ Research Program. The CLD codes (Table S1) for Hepatitis B and Hepatitis C were obtained from a previous report [21], and the codes for NAFLD and ALD were obtained from Phenotype KnowledgeBase (PheKB, http://phekb.org) [22]. Those patients who reported multiple CLDs were counted only once in our CLD cohort. However, some patients have multiple CLD subtypes, and the percentage was calculated based on the total number of patients reported in each CLD subtype.

Since the ‘All of Us’ researcher workbench does not require Institutional Review Board approval for each research project, as per the ‘Data User Code of Conduct’, the Resource Access Board of the ‘All of Us’ Research Program approved the study proposal. As per the ‘All of Us’ safe data sharing policy, categories with <20 participants were not included in the analysis. Any participant-level data involving fewer than 20 individuals are presented in tables as ≤ 20. A flow chart depicting our method of the ‘All of Us’ study population selection and confounding factor analysis is given in Supplementary Figure S1.

### 2.2. Confounding Factors Scoring

#### 2.2.1. Lack of Immunization

The CDC, Advisory Committees on Immunization Practices (ACIP), and the American Association for the Study of Liver Diseases (AASLD) recommend vaccination in CLD against Hepatitis A virus (HAV), Hepatitis B vaccine (HBV), influenza, pneumococcus, herpes zoster, tetanus-diphtheria-pertussis (TDaP), and SARS-CoV-2 [23]. Among the suggested vaccines, the herpes zoster vaccine was removed from this analysis because of insufficient data in CLD patients, and SARS-CoV-2 vaccination status was not included because the CLD data of this study were reported before 2019. The HAV, HBV, Pneumococcal, Influenza, and TDaP vaccination status [23] were obtained from the ‘drug exposures’ category of the ‘All of Us’ data. Based on these five vaccination status data, the ‘lack of immunization’ was calculated as an additive score ranging from 0 to 5 (Table S2). According to this scoring, 0 represents the best condition (all selected vaccinations), and 5 represents the worst condition (lack of all selected immunizations).

#### 2.2.2. Comorbidities

The comorbidity factors of CLD were selected according to the previous publications [24,25]. Chalasani et al. reported that obesity, type 2 diabetes, dyslipidemia, hypothyroidism, sleep apnea, metabolic syndrome (MetS), and polycystic ovary syndrome are strong predictors of CLD, especially in NAFLD [24]. However, since this study population includes both male and female participants, polycystic ovary syndrome was not considered for analysis. Similarly, dyslipidemia and MetS were excluded from the comorbidity factor analysis as dyslipidemia patients and participants who qualified for the criteria for MetS [26] were insufficient in the CLD cohort. Uhanova et al. suggested hypertension, type 2 diabetes, and obesity as the critical comorbid conditions of CLD [25]. Thus, the level of comorbidities was calculated based on five comorbidities: obesity (BMI > 30), type 2 diabetes, hypertension, sleep apnea, and hypothyroidism. For obesity, BMI was extracted from the ‘physical measurement data’, and type 2 diabetes, hypertension, sleep apnea, and hypothyroidism data were obtained from the ‘conditions’ category. The score was calculated from 0 (none) to 5 (all) depending on the number of comorbidities reported in a patient (Table S3).

#### 2.2.3. SES Barrier

The composite score for the SES factors was calculated based on five barriers, including education, income, insurance, housing, and employment status, as reported previously [27,28]. The selected questions for SES factors are listed in Table S4. The SES barrier score (0–5) was calculated depending on the number of reported individual barriers (Table S4). For comparison with non-CLD participants, SES barrier scores of CLD participants were compared to their age, gender, and race/ethnicity-matched non-CLD controls with 0 comorbidities.

### 2.3. Tumor Metabolic Factor Analysis

Since HCC is the most common form of LC accounting ~90% of cases [29], to avoid intrinsic variation, we only considered HCC as LC. A previous report comparing the metabolism of LC patients and healthy population reported an increased purine metabolite, hypoxanthine, in the urine of LC patients compared to the healthy volunteers [30]. Thus, we analyzed mRNA expressions of the purine metabolic pathway between normal liver and LC tissues using the GENT2 database [20]. GENT2 provided a user-friendly search platform for gene expression patterns across different normal and tumor tissues compiled from public gene expression datasets. For the validation studies, previously published gene expression data from LC and benign tissues (GSE77314, GSE184733, and GSE202853) were used. Confirmation studies of purine pathway-related genes in different race and ethnic groups and clinical survival analyses were performed using the Liver Hepatocellular Carcinoma transcriptomic dataset (TCGA, PanCancer Atlas) [31] from the cbioportal.

### 2.4. Statistical Analysis

Descriptive statistics were utilized to characterize the study population, encompassing all demographic variables and variables of interest. Multivariable logistic regression was employed to calculate the odds ratio (OR) and 95% confidence intervals (CIs) for various CLD incidence risks across different racial groups. This analysis was adjusted for ’age at the survey’, sex, and insurance status. For validation (Figure S10), each confounding factor (lack of immunization, comorbidities, or SES barrier) was analyzed after adjusting to age at diagnosis, gender, and the other two factors. All statistical analyses were conducted using ‘finalfit’ [32] and ‘freqtables’ [33] R packages in R studio integrated into the Jupyter notebook within the ‘All of Us’ workbench, with a significance level set at alpha 0.05. For the comparison between paired tumor and normal liver tissues, a paired *t*-test was used with a significance level of *p* < 0.05. The overall survival (OS) was analyzed using the clinical survival data reported in the TCGA database. According to the gene expression in the tumor, patients were classified as ’High’ and ’Low’ using the median cutoff. Kaplan–Meier OS was analyzed using the ’survival package’ in the R program.

## 3. Results

### 3.1. Characteristics of CLD Patients

**Figure 1:**
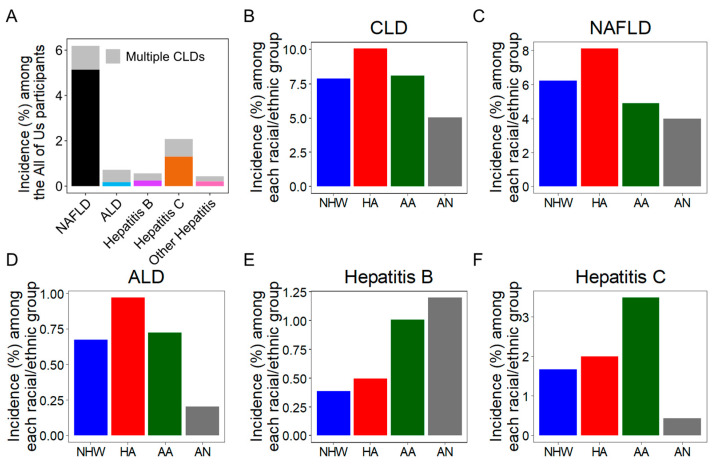
Distribution of CLD in the ‘All of Us’ database: (**A**). The incidence of CLD subtypes among all the participants enrolled in the ‘All of Us’ database irrespective of reported years of incidence (1980–2022). The gray color represents the participants with more than one CLD subtype. (**B**). The percentage of CLD reported within each ethnic/racial population in the ‘All of Us’ database. (**C**–**F**). The percentage of NAFLD (**C**), ALD (**D**), Hepatitis B (**E**), and Hepatitis C (**F**) reported within each ethnic/racial population in the ‘All of Us’ database.

Analysis of the ‘All of Us’ database identified 33767 CLD patients. The total number of CLD was calculated based on the unique patient ID. In the CLD subgroup analysis, there were 25404 NAFLD (6.2%), 2955 ALD (0.23%), 2314 Hepatitis B (0.25%), 8537 Hepatitis C (1.4%), and other Hepatitis (0.15%) (Figure 1A) patients. The demographic characteristics of CLD patients are described in Table S5. Overall, there were more female CLD patients compared to males. This is probably because of a relatively high proportion of female NAFLD cases, which is a major subgroup in the database (Table S5).

### 3.2. Racial Disparity Among the CLD Cohort

Among the ‘All of Us’ participants, approximately 10.03% of the HA, 8.1% of the AA, 7.9% of the NHW, and 5.0% of the AN population reported CLD (Figure 1B), indicating a comparatively higher prevalence of CLD among HA and AA populations compared to the NHW. Among CLD subgroups, NAFLD (Figure 1C) and ALD (Figure 1D) were more prevalent in the HA population, and Hepatitis C was more prevalent in the AA population (Figure 1F). Though the total number of Hepatitis B cases was low, the AN population showed the highest prevalence of Hepatitis B, followed by the AA population (Figure 1E). All these suggest a considerable over-representation of CLD subgroups among the ethnic/racial minority compared to the NHW population.

**Figure 2:**
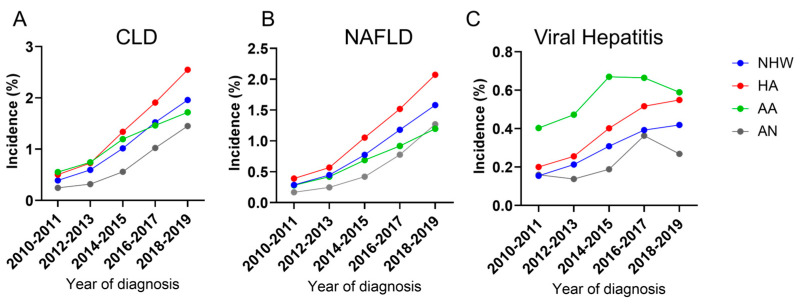
**(A**). The year-wise incidence of total CLD in different racial/ethnic groups over 10 years (2010–2019). (**B**,**C**). The year-wise incidence of NAFLD (**B**) and viral Hepatitis (**C**) in different racial/ethnic groups over 10 years. The incidence is calculated within the total population in each racial group present in the ‘All of Us’ data.

Overall, there was a consistent upward trend (2010–2019) in the prevalence of CLD cases among all racial groups reported in the ‘All of Us’ data analyzed (Figure 2A). In the CLD subgroup analysis, due to insufficient data, ALD was excluded from year-wise analysis and Hepatitis B, Hepatitis C, and other Hepatitis cases were combined as viral Hepatitis. The year-wise trend shows that NAFLD continuously increased in all four racial subgroups (Figure 2B). The difference in NAFLD incidence observed between AA and AN populations in the earlier years was not evident recently. Moreover, a recent downward trend was observed in the prevalence of viral Hepatitis among AA and AN (Figure 2C) populations.

### 3.3. Confounding Factors Associated with Racial Disparity

**Figure 3:**
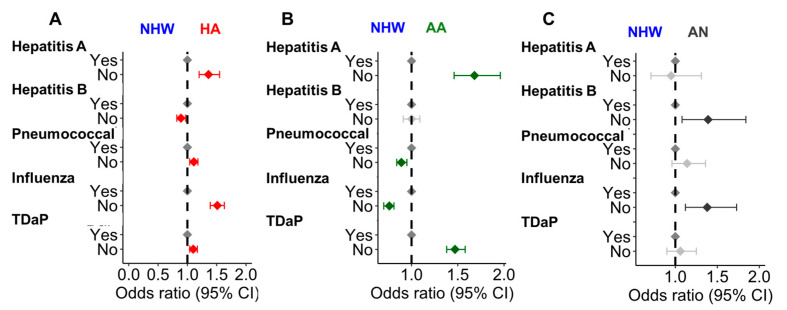
Age at diagnosis, gender, and insurance status adjusted OR of the lack of immunization in HA (**A**), AA (**B**), and AN (**C**) CLD patients compared to NHW CLD patients. The lack of immunization scores is not plotted due to <20 AN participants in some categories. The red, green, and dark gray colors represent significant ORs (*p* < 0.05) in HA, AA, and AN patients, respectively, compared to the NHW population. The light gray points represent the non-significant change (*p* > 0.05) in the ORs.

Lack of immunization: Hepatitis B, Hepatitis A, Pneumococcal, Influenza, and TDaP were evaluated among CLD patients because they are part of the recommended vaccines for CLD patients by the ACIP [23]. While 51.0% of the patients did not report receiving any of these vaccines, only <1% reported receiving all five vaccines (Table S6). The prevalence of Hepatitis A, Hepatitis B, Pneumococcal, Influenza, and TDaP-vaccinated CLD patients were 5.4%, 11.9%, 32.9%, 18.3%, and 30.9%, respectively (Table S6). Analysis of the racial/ethnic disparity in the vaccination status of CLD patients showed a lower prevalence of Hepatitis A and TDaP vaccination among HA and AA patients compared to the NHW (Figure 3A,B). However, in the AN CLD population, Hepatitis B and Influenza vaccines were lower than the NHW population (Figure 3C). This suggests that the lack of individual immunization varies among different racial subgroups. Since the number of the AN population in some of the groups was <20, the overall lack of immunization score was not calculated among the CLD population. CLD subgroup analysis suggests that the overall lack of immunization score was significant only in the NAFLD HA population (Figure S2A).

**Figure 4:**
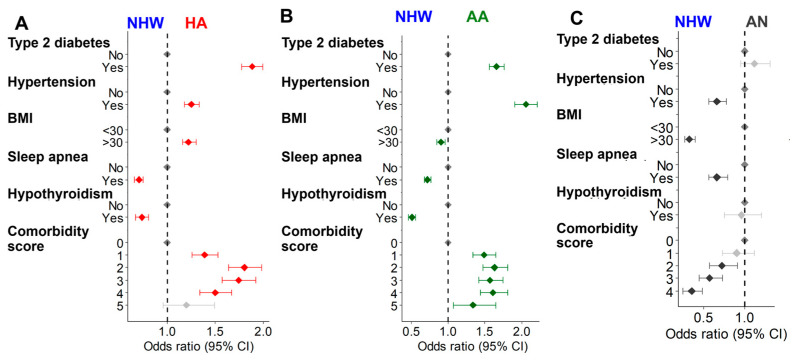
Age at diagnosis and the gender-adjusted OR of comorbidities in HA (**A**), AA (**B**), and AN (**C**) CLD patients compared to the NHW population. The red, green, and dark gray colors represent significant ORs (*p* < 0.05) in HA, AA, and AN patients, respectively, compared to the NHW population. The light gray points represent the non-significant change (*p* > 0.05) in the ORs.

Comorbidities: Many ‘All of Us’ CLD patients reported having the analyzed comorbidities including type 2 diabetes (40.6%), hypertension (69.4%), obesity (BMI > 30) (53.1%), sleep apnea (31.9%), and hypothyroidism (12.0%) (Table S7). Consequently, only a low percentage (14.3%) of CLD patients reported without any of these five comorbidities (Table S7). While the difference in individual comorbidities compared to the NHW population varied among racial/ethnic groups, both HA and AA CLD patients showed a significantly increased OR of type 2 diabetes and hypertension (Figure 4A,B). However, most of the individual comorbidities were significantly less in the AN CLD patients (Figure 4C). BMI was significantly increased only in the HA CLD population (Figure 4A). However, sleep apnea was significantly more prevalent in the NHW population compared to the minority populations. HA and AA CLD populations showed significantly less prevalence of hypothyroidism compared to NHW patients. However, the overall comorbidity score suggests that CLD patients having multiple comorbidities are significantly higher among HA and AA patients and lower in the AN population than NHW patients (Figure 4). Similarly, the overall comorbidity score of CLD subtypes was significantly high in HA and AA populations (Figure S3).

**Figure 5:**
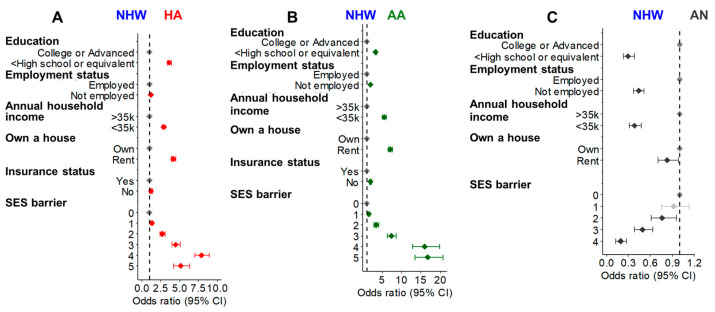
Age at diagnosis and the gender-adjusted OR of SES barriers in HA (**A**), AA (**B**), and AN (**C**) CLD patients compared to the NHW patients. The SES barrier ‘5’ cannot be plotted in the AN population because of < 20 participants. The red, green, and dark gray colors represent significant ORs (*p* < 0.05) in HA, AA, and AN patients, respectively, compared to the NHW patients. The light gray points represent the non-significant change (*p* > 0.05) in the ORs.

Socioeconomic barriers: Multivariable logistic regression analysis was used to evaluate the five primary SES barriers (education, employment status, annual household income, housing, and insurance) that influence overall living status [27,28]. We used SES score 0 (no SES barrier) to 5 (all 5 SES barriers) for the analysis. In all ethnic groups (except AN), these SES barrier scores were higher among the CLD cohort compared to their age, gender, and race/ethnicity-matched non-CLD controls that lack common comorbidities [obesity (BMI > 30), type 2 diabetes, hypertension, sleep apnea, and hypothyroidism] (Figure S4A–C). Interestingly, most SES barriers were not significant in the AN CLD population compared to non-CLD (Figure S4D). Strikingly, the racial disparity analysis suggests that all the SES barrier scores (1–5) were significantly increased among HA and AA CLD patients compared to the NHW patients (Figure 5A,B). However, in AN CLD, the SES barriers were lower compared to NHW (Figure 5C). In CLD subgroup analysis, SES barriers were higher in HA and AA NAFLD and viral Hepatitis patients (Figure S5). These results highlight the existing SES barriers in HA and AA CLD patients that could significantly influence their disease outcome.

### 3.4. LC Patients in ‘All of Us’ Database

Since CLD is a major predisposing factor for LC [2,6,7] and advanced CLD can significantly elevate the risk of developing LC [10], we then analyzed the LC patients in the ‘All of Us’ data. Overall, 556 ‘All of Us’ participants reported LC. As expected, the overlapping of patient IDs among the LC and CLD patients suggests that 92% of LC patients also reported CLD (Figure S6A). The racial distribution of LC patients in the ‘All of Us’ database suggests that LC is more prevalent in HA population (Figure S6B). Due to the limited number of LC patients in the ‘All of Us’ database, further subgroup analysis of confounding factors was not feasible in the LC population.

Though several biological factors reported in LC patients, only limited studies reported about its racial disparity. So we analyzed tumor intrinsic biological factors that show disparity in LC patients. Since LC is often associated with metabolic dysregulation [34], we focused on the potential role of metabolism in LC patients.

### 3.5. Purine Metabolism Is Activated in LC

From a metabolic point of view, a previous study that compared the urine samples from healthy controls and LC patients reported that hypoxanthine is the only metabolite that is significantly upregulated, and creatinine and carnitines are the significantly downregulated metabolites in LC patients [30]. Hypoxanthine is one of the downstream metabolites of the purine catabolism pathway in humans, where the oxidation of hypoxanthine to xanthine by xanthine dehydrogenase (XDH) is essential in converting xanthine to uric acid as the end product (Figure S7).

**Figure 6:**
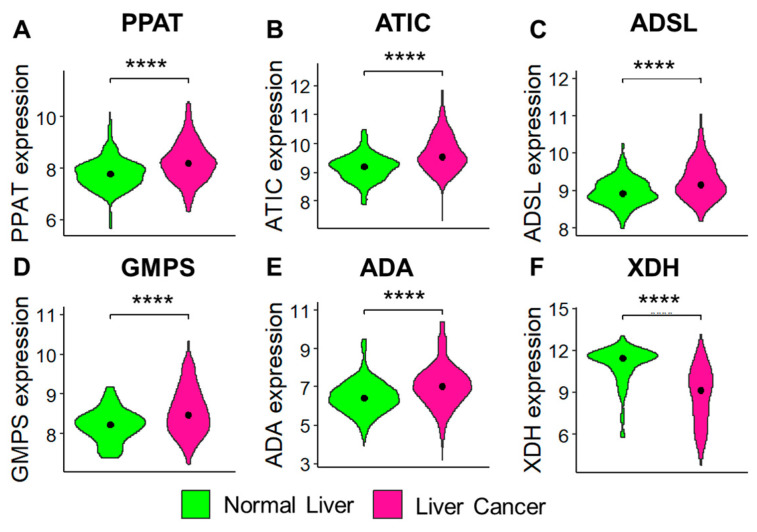
(**A–F**) Violin plots of GENT2 data comparing the mRNA expression of purine metabolism-related genes (illustrated in Figure S7), PPAT (**A**), ATIC (**B**), ADSL (**C**), GMPS (**D**), ADA (**E**), and XDH (**F**) between the normal liver and LC tissue. **** represents *p* < 0.0001 by two-tailed *t*-test.

Since purine metabolism is vital in cancer progression, the genes associated with the purine metabolism pathway were analyzed in benign and LC tissues using the GENT2 database (Figure 6). The GENT2 database has gene expression patterns across different normal and tumor tissues compiled from public gene expression datasets [20]. GENT2 analysis suggests that most purine pathway genes (Figure S7) upstream of hypoxanthine were upregulated in the LC tissues compared to the normal liver tissues (Figure 6A–E). Interestingly, only XDH, which catalyzes hypoxanthine to uric acid, was downregulated in the tumor tissues (Figure 6F). This suggests that the metabolism of hypoxanthine is impaired in LC tissues compared to normal liver tissues.

### 3.6. Inhibition of XDH Is More Prevalent in AN LC

**Figure 7:**
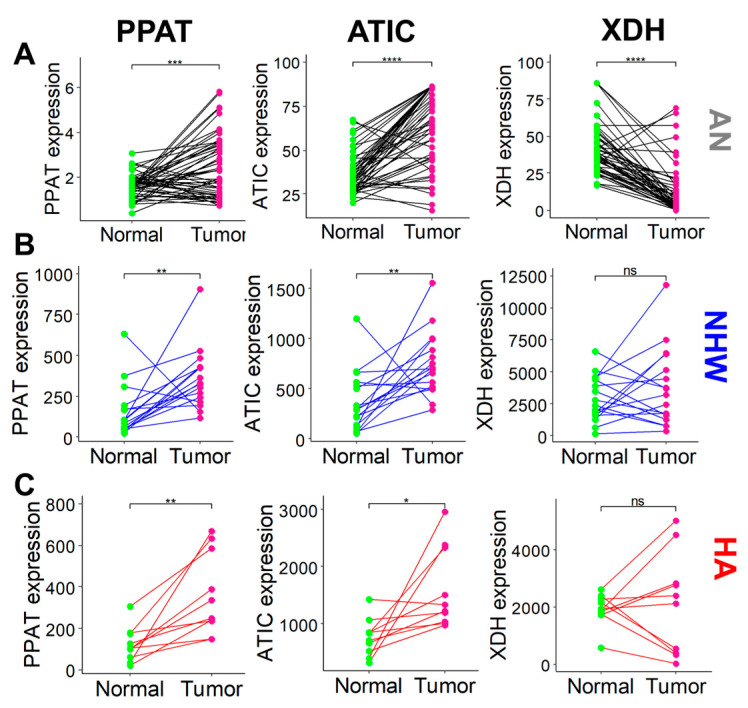
Gene expression data from normal and cancer tissues from LC patients from 50 pairs of AN patients (GSE77314) (**A**), 17 pairs of NHW patients (GSE184733) (**B**), and 10 pairs of HA patients (GSE202853) (**C**). *, **, ***, ****, and ns represent the *p* < 0.05, *p* < 0.01, *p* < 0.001, *p* < 0.0001 and *p* > 0.05 respectively by paired *t*-test.

These data were validated in different racial/ethnic groups using other published gene expression data from normal and LC tissues. GSE184733 contains gene expression data of 17 pairs of benign and LC tissues from NHW patients [35], GSE202853 includes data from 10 HA LC patients [36], and GSE77314 has 50 pairs of data from AN LC patients [37]. These transcriptomic data suggest that, as observed with the GENT2 database, most of the purine pathway genes are upregulated in the LC compared to the matching benign tissues irrespective of their racial/ethnic background (Figure 7). However, XDH was significantly downregulated in the LC tissues of only AN patients but not in NHW or HA patients (Figure 7). This suggests that the downregulation of the XDH enzyme in the LC may be critical for AN LC patients.

**Figure 8:**
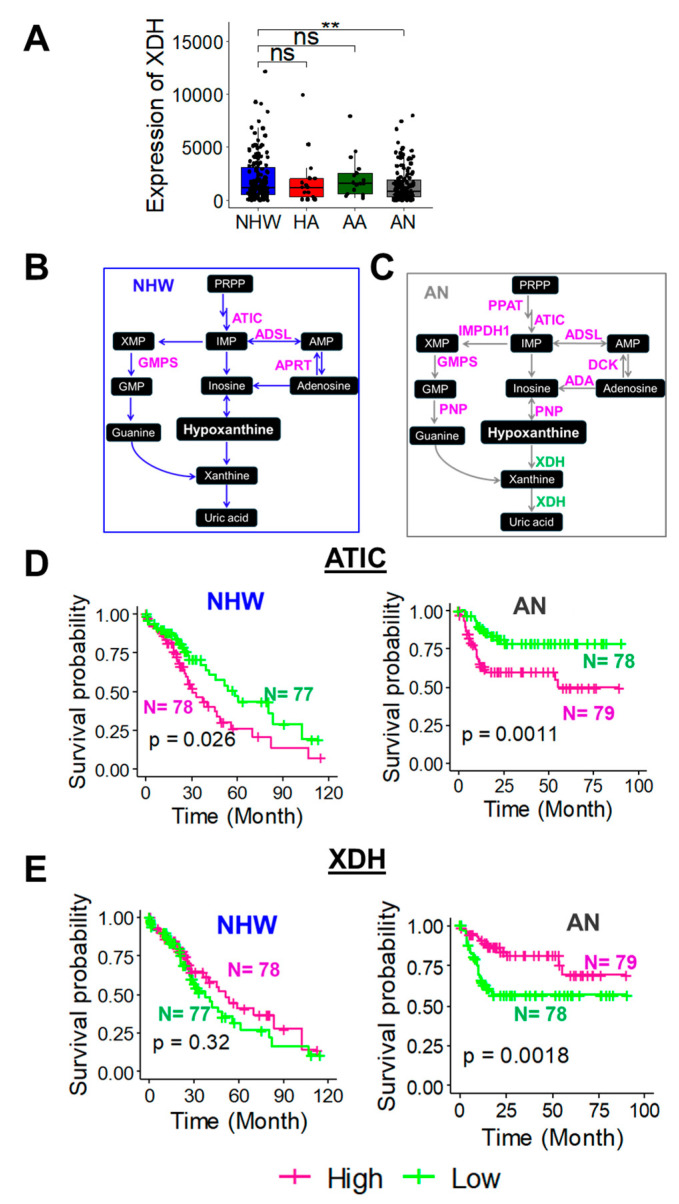
(**A**). The differential expression of XDH mRNA in LC among different racial/ethnic groups. (**B**,**C**). The enzymes involved in the purine metabolism pathway are analyzed in the TCGA data among NHW (**B**) and AN (**C**) LC patients. The pink color letters represent the enzymes associated with poor survival at higher mRNA expression. The green color letters represent the enzyme associated with poor survival at a lower mRNA expression. (**D**,**E**). Kaplan–Meier survival curve of the OS of ATIC (**D**) and XDH (**E**) mRNA in NHW and AN LC patients. Median mRNA expression separates high (pink) and low (green) sub-groups. ** and ns represent the *p* < 0.01 and *p* > 0.05 respectively by two-tailed *t*-test.

This observation was further confirmed in TCGA tumor data. As observed above, only in the AN patients, XDH was significantly downregulated in the LC tumors compared to the NHW patients (Figure 8A). However, HA or AA tumors did not show a significant downregulation of XDH compared to NHW patients, confirming that XDH downregulation has potential clinical significance in AN LC patients. To further understand the clinical significance of this finding, the overall survival (OS) of purine metabolism genes in different racial/ethnic LC patients were evaluated using the TCGA database. A medium cutoff point of gene expression suggested that the higher expression of most of the genes associated with the purine pathway upstream of hypoxanthine leads to the poor overall survival of LC patients (Figure 8B–D and Figures S8 and S9). However, the lower expression of XDH showed a significantly poor overall survival only in the AN LC patients (Figure 8E), further confirming the clinical significance of decreased XDH and increased hypoxanthine accumulation in AN LC patients.

## 4. Discussion

Overall, the analysis of biosocial factors using different databases suggests that social factors are more prevalent in HA and AA CLD patients. Similarly, a racial disparity exists in the hypoxanthine-regulated metabolic pathway among the AN LC population.

The incidence of LC has been steadily increasing over the past decades [38]. A recent study showed mechanistic and functional evidence supporting a causal relationship between human CLD and liver carcinogenesis [39]. Most of the LC patients are reported to have a history of CLD [6,7]. The ‘All of Us’ data analysis in this study also suggests that 92% of LC patients have CLD. Therefore, it is crucial to identify the factors that influence CLD, which is critical in understanding the biological and racial disparities that contribute to LC-related morbidities in under-represented minorities.

Biosocial determinants, representing the intersection of social and biological factors, can show how biosocial synergism contributes to disparities [15]. Previous studies suggest that promoting healthy lifestyles, improving access to healthcare, and early disease detection are crucial for reducing the burden of CLD and its complications on individuals and the healthcare system [40,41,42,43]. While it is difficult to identify a single root cause, it is essential to understand the complex interplay of factors contributing to racial disparities in CLD/LC for immediate attention to address and minimize the disparity [44]. However, existing population databases like the National Health and Nutrition Examination Survey (NHANES) have limitations in fully capturing the interplay of modifiable risk factors within the same populations [40]. Similarly, while the United Network for Organ Sharing (UNOS) provides data on healthcare access and utilization, it lacks information on the same population’s economic, educational, social, and health behaviors [40]. The evolvement of the ‘All of Us’ database, one of the largest diverse US cohorts with a significant inclusion of ethnic/racial minority populations, provides an excellent opportunity to address some of these currently unknown insights into racial disparity [45].

Minority populations face barriers to accessing healthcare services, including vaccination [46,47,48]. However, in this study, the lack of individual vaccination status differed among different minority groups. For example, while the AN population reported the highest prevalence of the Hepatitis B CLD subtype (Figure 1E), they also reported a significant lack of Hepatitis B vaccination status (Figure 3C). Similarly, while the HA population reported the highest prevalence of the NAFLD CLD subtype (Figure 1C), they also reported a significant lack of immunization scores in their NAFLD cohort (Figure S2A). SES factors, a lack of health insurance, and mistrust of the healthcare system can contribute to lower vaccination rates in this population [49]. Recent findings also suggest that, in patients with CLD, the coinfection of another acute disease (such as Hepatitis virus superinfection, influenza, and pneumococcal infection) may result in higher morbidity and mortality than in individuals without pre-existing liver disease [50,51,52]. Despite the potential benefits of vaccination and the solid recommendations from ACIP and AASLD [23], the vaccination rates in this vulnerable population remain below optimal levels [53]. This underscores the need for targeted interventions and specific vaccination campaigns within each under-represented population to reduce CLD and LC complications.

Previous reports suggest that individuals with comorbidities like obesity, type 2 diabetes, dyslipidemia, hypothyroidism, sleep apnea, MetS, and polycystic ovary syndrome are at a higher risk of developing CLD and/or HCC [24,54,55]. In this study, HA and AA CLD patients exhibited a higher prevalence of comorbidities, indicating the importance of holistic approaches to managing CLD and its associated conditions. Genetic and lifestyle factors including dietary habits are known to contribute to various comorbidities in minority populations [56,57]. However, the comorbidity scores were better in AN CLD patients. This observation aligns with the lower prevalence of overall CLD among the AN population compared to other racial/ethnic groups.

The higher SES barriers with HA and AA CLD patients observed in this study highlight the compounding effect of multiple socioeconomic disadvantages on the risk of developing CLD. A previous report conducted by Ohikere et al. [58] suggested an interconnection between ethnic minorities and low SES in the CLD population. A recent review by Kardashian et al. [43] on CLD suggests that racial disparities lead to delays in diagnosis, unequal access to therapy for viral Hepatitis and liver transplantation, as well as unequal health outcomes. However, as expected from the decreased CLD prevalence in AN population, the SES barriers were also lower in AN CLD patients compared to the NHW patients. Overall, these SES barriers in HA and AA CLD patients are particularly concerning, as they can lead to limited access to healthcare, delayed diagnosis, and the suboptimal management of CLD.

To further strengthen the data, we reanalyzed each confounding factor (the lack of immunization, comorbidities, or the SES barrier) after adjusting to age at diagnosis, gender, and the other two factors (Figure S10). For example, the OR for lack of immunization was adjusted to age at diagnosis, gender, comorbidities, and the SES barrier. Even in this stringent analysis, the HA and AA CLD population showed higher odds of all confounding factors than the NHW. Considering the close relationship between CLD and LC, the confounding factors identified for CLD patients may also contribute to the development and progression of LC among the under-represented population. However, due to the limited number of LC patients in the ‘All of Us’ data, confounding factor analysis was not feasible in this study.

In addition to these various modifiable risk factors, we must also consider the plausible non-modifiable risk factors, such as biological factors that affect cancer disparity, often through interactions with SES [15,59]. Moreover, lifestyle and SES can influence dietary choice, nutrient availability, and liver metabolism, leading to cancer risk [60,61]. Metabolism and metabolic reprogramming are critical to cancer development and progression [62]. Tumor cells rewire their metabolic pathways to maintain metabolic plasticity [63], sustain their growth, survive in unfavorable conditions, and crosstalk with the tumor microenvironment to promote tumor progression and metastasis [64]. Bidirectional communication exists between LC and metabolism. While metabolic disorders enhance the LC risk [3], LC can also modulate metabolic balance or cause metabolic dysfunction [16,29,34,65,66]. A few metabolites and their associated genes have been reported as biomarkers in LC patients [67,68,69]. However, there are only limited reports of any such metabolism-related biomarker regarding racial disparity in LC. Thus, analyzing the differential metabolic pathways contributing to LC is vital in understanding the ethnic/racial inequality among LC patients.

In this study, a significant association between purine metabolism and LC prognosis was observed, which is consistent with the earlier studies [70,71,72]. Altered purine metabolism, including hypoxanthine accumulation, can influence LC because it generates oxidative stress that causes inflammation and metabolic disturbances [73]. These stress factors can cause damage to the liver tissues and increase LC risk. Thus, hypoxanthine potentially serves as a biomarker for liver dysfunction or LC progression. The hypoxanthine metabolite of purine is reported to have a direct connection with some of the lifestyle factors, such as smoking and drinking [74].

XDH oxidizes hypoxanthine to xanthine and, subsequently, xanthine to uric acid [75]. Thus, XDH is considered as a rate-limiting enzyme in purine metabolism [76]. The decreased ROS production from low XDH activity that causes the reduced production of uric acid may seem protective [77]. However, ROS is a double-edged sword in cancer signaling. While excessive ROS can cause DNA mutations and cancer progression [78], ROS also induces several signaling pathways that can trigger apoptosis in cancer cells [79]. As a ROS scavenger, uric acid functions as an antioxidant [80]. Thus, the reduction in ROS might impact the balance between cell survival and death in LC tissues. The low XDH-mediated reduction in uric acid production could suppress this antioxidant defense and induce oxidative stress [81]. Such induction of oxidative stress can cause tissue injury and various diseases, including LC. However, so far, no studies reported the role of XDH among different racial/ethnic populations.

Our analysis found that low XDH expression in the tumors leads to poor prognosis in AN LC patients but not in other ethnic/racial subgroups. This low XDH-driven poor prognosis further confirms that a possible lack of uric acid generation or activation of alternative pathways by the downregulation of XDH is critical in the progression of AN LC. Identifying this race-specific activation of alternative pathways requires more extensive gene expression and mechanistic studies. LC is a significant health concern in AN populations due to the combination of various confounding factors. These factors include genetics, healthcare access, a high prevalence of hepatitis, and exposure to different environmental toxins [82,83,84]. The complexity of the AN diet can have both risk factors and protective elements to LC. Traditional AN diets are rich in plant-based ingredients [85], and that may provide some protection for the AN population from liver diseases. However, high salt intake [86], alcohol consumption [87], and aflatoxin exposure [88] can increase LC risk. Moreover, the recent adoption of more Westernized eating patterns by the AN population can also enhance their LC risk [89]. Understanding the role of the purine pathway and the increased accumulation of hypoxanthine in AN tumors could help to develop new strategies for preventing and treating LC among the AN population.

## 5. Strength and Limitations

This study’s major strengths are identifying critical confounding and SES factors that influence the risk and outcome of CLD/LC patients. Moreover, we identified a critical metabolic rate-limiting enzyme with clinical significance in AN LC patients. In addition, this study utilized the ‘All of Us’ data that include a higher proportion of geographically and ethnically diverse minority populations. These more extensive and diverse data help deepen the current understanding of health disparities among CLD/LC patients. Overall, this study provides a biosocial interconnection that leads to the development and progression of CLD and LC in the minority population. Identifying a racial disparity in the XDH expression in AN LC tumors is unique in further understanding the alternative pathways activated by the accumulation of hypoxanthine and its contribution to the progression of LC in AN patients.

However, in our cohort selection, there is the possibility of missing some patients due to the ICD codes that we used. Moreover, some CLD subtypes and ethnic groups, like ANs, were not considered for the downstream analysis due to an insufficient sample number (<20) in some subgroups. Since the ‘All of Us’ data are from self-reported questionnaires from the participants at the time of the survey, there is the possibility of missing information. Though HCC represents ~90% of LC, some databases did not specify if all cohorts are from HCC. Thus, we considered them as LC. Though TCGA data have information about a few cholangiocellular (LC subtype) patients, we could not analyze them separately due to a lack of power. Since the TCGA only has a low number of LC data from ethnic minority groups like AA and HA patients, the survival factor observed in this population may require analysis in a larger cohort to obtain conclusive results.

## 6. Conclusions

This study’s findings contribute to the increasing evidence of disparities among ethnic/racial minority CLD populations in the USA, highlighting the need for multifaceted interventions to address the low SES and high comorbidities among HA and AA populations. This study also identified decreased XDH expression in LC that can enhance hypoxanthine levels in the tumor as a critical metabolic alteration with a clinical significance in AN LC patients. Targeting these biosocial factors among individual racial/ethnic groups is critical for a better, equitable healthcare system that improves outcomes for minority populations affected by CLD and LC.

## Data Availability

These data were derived from the following resources available in the public domain: All of Us Research Workbench: https://workbench.researchallofus.org/ (accessed on 3 March 2024); TCGA: www.cbioportal.org; GENT2: http://gent2.appex.kr (accessed on 16 August 2024).

## Supplementary Materials

The following supporting information can be downloaded at: https://www.mdpi.com/article/10.3390/cancers17050844/s1, Figure S1: Flow chart of the All of Us data analysis; Figure S2: Age at diagnosis, gender, and insurance status adjusted Odds Ratio (OR) of lack of immunization in HA and AA CLD patients compared to NHW patients; Figure S3: Age at diagnosis and gender-adjusted OR of comorbidities in HA and AA patients compared to NHW patients; Figure S4: Age at diagnosis and gender-adjusted OR of SES barriers in HA (A), AA (B), AN (C), and NHW (C) CLD patients compared to their age, gender and race-matched non-CLD controls without any obesity (BMI >30), Type 2 diabetes, hypertension, sleep apnea, and hypothyroidism (comorbidity score ‘0’); Figure S5: Age at diagnosis and gender-adjusted OR of SES barriers in HA and AA patients compared to NHW; Figure S6: (A) The overlap of CLD and LC patients in ‘All of Us’ data. (B) Racial distribution of ‘All of Us’ participants reported LC; Figure S7: Purine Metabolism Pathway metabolites and enzymes; Figure S8: (A,B) The enzymes involved in the purine metabolism pathway were analyzed in the TCGA data among HA (A) and AA (B) LC patients. The pink color letters represent the enzymes associated with poor survival at higher mRNA expression. The black color letters represents enzymes with no prognostic significance. (C,D) Kaplan-Meier survival curve of OS of ATIC (C) and XDH (D) mRNA in HA and AA LC patients. Median mRNA expression separated high (pink) and low (green) sub-groups; Figure S9: Kaplan-Meier survival curve of OS of purine metabolism genes in NHW (A) and in AN (B) LC patients. Median mRNA expression separated high (pink) and low (green) sub-groups; Figure S10: The OR of the three confounding factors (lack of immunization, comorbidities, and SES barrier) among (A) HA and (B) AA CLD, NAFLD, and viral hepatitis patients compared to NHW. Each confounding factor was adjusted to Age at diagnosis, gender, and the other two factors; Table S1: ICD-9 and ICD-10 diagnosis codes used for cohort selection; Table S2: Definitions and classifications of immunization or vaccination status analyzed; Table S3: Definitions and classifications of comorbidities analyzed in the study; Table S4: Definitions and classifications of SES barriers; Table S5: Characteristics of all CLD cohort according to different subtypes of CLD; Table S6: Characteristics of all CLD cohort according to immunization; Table S7: Characteristics of all CLD cohort according to comorbidities.
