# Supplementary material for: Biosocial Determinants of Health Among Patients with Chronic Liver Disease and Liver Cancer
Source: Cancers (Basel). 2025 Feb 28;17(5):844. doi: 10.3390/cancers17050844 (PMC11898429; doi:10.3390/cancers17050844)
Supplement: Supplementary file 1 [file cancers-17-00844-s001.zip › cancers-3405996-supplementary.pdf]

Biosocial determinants of health disparities among racial/ethnic minorities with chronic liver disease and liver cancer

Tagari Samanta[1], Jun Hyoung Park[1], and Benny A. Kaipparettu[1]

[1]Department of Molecular and Human Genetics, Baylor College of Medicine, Houston, TX, USA.

*Corresponding author: Benny Abraham Kaipparettu, Department of Molecular and Cellular Biology, Baylor College of Medicine, Houston, TX, USA (kaippare@bcm.edu)

Table S1: ICD-9 and ICD-10 diagnosis codes used for cohort selection

ICD 9/10 code	Description
**Non-alcoholic fatty liver disease**	
571.5	Cirrhosis of liver without mention of alcohol
571.8	Other chronic nonalcoholic liver disease
571.9	Unspecified chronic liver disease without mention of alcohol
K75.81	Nonalcoholic steatohepatitis
K76.0	Fatty (change of) liver, not elsewhere classified
K76.9	Liver disease, unspecified
**Alcoholic liver disease**	
571.0	Alcoholic fatty liver
571.1	Acute alcoholic hepatitis
571.2	Alcohol cirrhosis liver
571.3	Alcoholic liver damage, unspecified
K70	Alcoholic liver disease
K70.0	Alcoholic fatty liver
K70.1	Alcoholic hepatitis
K70.10	Alcoholic hepatitis without ascites
K70.3	Alcoholic cirrhosis of liver
K70.30	Alcoholic cirrhosis of liver without ascites
K70.31	Alcoholic cirrhosis of liver with ascites
K70.11	Alcoholic hepatitis with ascites
K70.9	Alcoholic liver disease, unspecified
**Hepatitis C**	
070.41	Acute hepatitis C with hepatic coma
070.44	Chronic hepatitis C with hepatic coma
070.51	Acute hepatitis C without mention of hepatic coma
070.54	Chronic hepatitis C without mention of hepatic coma
070.70	Unspecified viral hepatitis C without hepatic coma
070.7	unspecified viral hepatitis C without hepatic coma
070.71	Unspecified viral hepatitis C with hepatic coma
B17.11	Acute hepatitis C with hepatic coma
B19.20	Unspecified viral hepatitis C without hepatic coma
B18.2	Chronic viral hepatitis C
B19.2	Unspecified viral hepatitis C
B19.21	Unspecified viral hepatitis C with hepatic coma
**Hepatitis B**	
070.20	Viral hepatitis B with hepatic coma, acute or unspecified, without mention of hepatitis delta
070.2	Acute or unspecified viral hepatitis B with hepatic coma, without mention of hepatitis delta
070.21	Viral hepatitis B with hepatic coma, acute or unspecified, with hepatitis delta
070.22	Viral hepatitis B with hepatic coma, chronic, without mention of hepatitis delta
070.23	Viral hepatitis B with hepatic coma, chronic with hepatitis delta
070.3	Viral hepatitis b without mention of hepatic coma
070.30	Viral hepatitis B without mention of hepatic coma, acute or unspecified, without mention of hepatitis delta
070.31	Viral hepatitis B without mention of hepatic coma, acute or unspecified, with hepatitis delta
070.32	Viral hepatitis B without mention of hepatic coma, chronic, without mention of hepatitis delta
070.33	Viral hepatitis B without mention of hepatic coma, chronic with hepatitis delta

070.42	Hepatitis delta without mention of active hepatitis B disease with hepatic coma
070.52	Hepatitis delta without mention of active hepatitis B disease or hepatic coma
B16	Acute hepatitis B
B16.0	Acute hepatitis B with delta-agent (coinfection) with hepatic coma
B16.2	Acute hepatitis B without delta-agent with hepatic coma
B16.1	Acute hepatitis B with delta-agent (coinfection) without hepatic coma
B16.9	Acute hepatitis B without delta-agent and without hepatic coma
B18.0	Chronic viral hepatitis B with delta-agent
B18.1	Chronic viral hepatitis B without delta-agent
B19.1	Unspecified viral hepatitis B
B19.11	Unspecified viral hepatitis B with hepatic coma
B19.10	Unspecified viral hepatitis B without hepatic coma
	**Viral Hepatitis other**
070.5	Other specified viral hepatitis without mention of hepatic coma
070.53	Hepatitis E without mention of hepatic coma
070.59	Other specified viral hepatitis without mention of hepatic coma
070.4	Other specified viral hepatitis with hepatic coma
070.43	Hepatitis E with hepatic coma
070.49	Other specified viral hepatitis with hepatic coma
B19	Unspecified viral hepatitis
070	viral hepatitis
070.0	Hepatitis A with hepatic coma
070.1	Hepatitis A without coma
070.6	Unspecified viral hepatitis with hepatic coma
070.9	Hepatitis, viral, NOS
B15.9	Hepatitis A without hepatic coma
B18	chronic viral hepatitis
B17.8	Other specified acute viral hepatitis
B17.9	Acute viral hepatitis that is unspecified
573.1	Hepatitis in viral diseases classified elsewhere
B18.9	Chronic viral hepatitis that is unspecified
B18.8	Other chronic viral hepatitis
B19.9	Unspecified viral hepatitis without hepatic coma

Table S2: Definitions and classifications of immunization or vaccination status analyzed

**Definitions and Classifications of Immunization or vaccination status**	
**Immunization or vaccination**	Description
**Hepatitis A**	Dichotomized into “Yes" (coded as 0) and “No" (coded as 1)
**Hepatitis B**	Dichotomized into “Yes" (coded as 0) and “No" (coded as 1)
**Pneumococcal**	Dichotomized into “Yes" (coded as 0) and “No" (coded as 1)
**Influenza**	Dichotomized into “Yes" (coded as 0) and “No" (coded as 1)
**TDaP (Tetanus, Diphtheria and Pertussis)**	Dichotomized into “Yes" (coded as 0) and “No" (coded as 1)
**Lack of Immunization Composite Score: Sum of Hepatitis A, Hepatitis A, Pneumococcal, Influenza, and TDaP vaccination status**	
**0**	No lack of immunization
**1**	Lack of one immunization
**2**	Lack of two immunization
**3**	Lack of three immunization
**4**	Lack of four immunization
**5**	Lack of five immunization

Table S3: Definitions and classifications of comorbidities analyzed in the study

Definitions and classifications of comorbidities	
**Comorbidities**	Description
**Type 2 Diabetes**	Dichotomized into “Yes" (coded as 1) and “No" (coded as 0)
**Hypertension**	Dichotomized into “Yes" (coded as 1) and “No" (coded as 0)
**Obesity (BMI 30+)**	Dichotomized into “Yes" (coded as 1) and “No" (coded as 0)
**Sleep apnea**	Dichotomized into “Yes" (coded as 1) and “No" (coded as 0)
**Hypothyroidism**	Dichotomized into “Yes" (coded as 1) and “No" (coded as 0)
**Comorbidities Composite Score: Sum of type 2 diabetes, hypertension and Obesity status**	
**0**	No comorbidities
**1**	One comorbidity
**2**	Two comorbidities
**3**	Three comorbidities
**4**	Four comorbidities
**5**	Five comorbidities

Table S4: Definitions and classifications of SES barriers

Questions asked from All of Us 'The basics' survey as used in a previous study	Abbreviated terms used in Figure 5	Scoring
What is the highest grade or year of school you completed?	Education	Dichotomized into "college or more" (coded as 0) and "≤ high school or equivalent" (coded as 1)
What is your annual household income from all sources?	Annual House-hold income	Dichotomized into "≥35K" (coded as 0) and "<35K" (coded as 1)
Are you covered by health insurance or some other kind of health care plan?	Insurance Status	Dichotomized into "Yes" (coded as 0) and "No" (coded as 1)
Do you own or rent the place where you live?	Own a house	Dichotomized into "owning a home" (coded as 0) and "rent/another arrangement" (coded as 1)
What is your current employment status?	Employment status	Dichotomized into "employed" (coded as 0) and "not employed" (coded as 1)
**SES Composite Score: Sum of Annual House-hold income, Education, Insurance status, Housing and Employment status**		
0		No SES barriers present
1		One SES barrier present
2		Two SES barriers present
3		Three SES barriers present
4		Four SES barriers present
5		Five SES barriers present

Table S5: Characteristics of all CLD cohort according to different subtypes of CLD

Table S5: Characteristics of all CLD cohort according to different subtypes of CLD					
	**NAFLD**	**ALD**	**Hepatitis B**	**Hepatitis C**	**CLD***
**Age at diagnosis, Mean (SD) in year**					
Mean (SD)	52.8 (13.8)	51.6 (11.8)	50.9 (13.0)	49.0 (12.1)	51.8 (13.7)
**Age group, Mean (SD) in year**					
18-44	6672 (26.4)	754 (25.6)	692 (30.1)	2841 (33.3)	9650 (28.7)
45-64	13205 (52.2)	1824 (61.8)	1258 (54.7)	4948 (58.0)	17592 (52.3)
65+	5426 (21.4)	372 (12.6)	348 (15.1)	735 (8.6)	6387 (19.0)
**Gender, n(%)**					
Female	15335 (60.4)	1092 (37.0)	1026 (44.3)	3464 (40.6)	18780 (55.6)
Male	9519 (37.5)	1787 (60.5)	1210 (52.3)	4789 (56.1)	14161 (41.9)
Others	550 (2.2)	76 (2.6)	78 (3.4)	284 (3.3)	826 (2.4)
**Insurance status, n(%)**					
Yes	23793 (96.6)	2672 (94.5)	2132 (95.7)	7522 (92.4)	31167 (95.6)
No	833 (3.4)	155 (5.5)	96 (4.3)	615 (7.6)	1441 (4.4)
**Education, n(%)**					
College or Advanced	16524 (67.2)	1445 (51.2)	1256 (57.0)	3406 (42.1)	20208 (62.2)
<High school or equivalent	8058 (32.8)	1375 (48.8)	947 (43.0)	4676 (57.9)	12305 (37.8)
**Annual household income, n(%)**					
>35k	10508 (53.9)	699 (32.8)	652 (37.5)	1216 (19.0)	12073 (46.9)
<35k	8982 (46.1)	1429 (67.2)	1087 (62.5)	5169 (81.0)	13695 (53.1)
**Own a House, n(%)**					
Own	11375 (51.8)	786 (34.2)	695 (36.2)	1453 (24.0)	13096 (47.0)
Rent	10582 (48.2)	1510 (65.8)	1225 (63.8)	4611 (76.0)	14769 (53.0)
**Employment Status, n(%)**					
Employed	9119 (37.3)	677 (24.0)	652 (29.6)	1550 (19.2)	11024 (34.1)
Not employed	15343 (62.7)	2139 (76.0)	1550 (70.4)	6520 (80.8)	21331 (65.9)
**Racial/Ethnic groups, n(%)**					
NHW	13644 (57.0)	1480 (53.2)	850 (39.5)	3695 (46.6)	17355 (54.8)
HA	5974 (25.0)	717 (25.8)	365 (17.0)	1483 (18.7)	7417 (23.4)
AA	3750 (15.7)	557 (20.0)	771 (35.8)	2683 (33.9)	6227 (19.6)
AN	551 (2.3)	28 (1.0)	165 (7.7)	60 (0.8)	692 (2.2)
* The total number of CLD is calculated based on the unique patient ID. Those patients who reported multiple CLDs were counted only once.					

Table S6: Characteristics of all CLD cohort according to immunization

**Table S6:** Characteristics of all CLD cohort according to immunization					
	**NAFLD**	**ALD**	**Hepatitis B**	**Hepatitis C**	**CLD**[*]
**Hepatitis A vaccine, n(%)**					
Yes	1357 (5.3)	272 (9.2)	208 (9.0)	563 (6.6)	1832 (5.4)
No	24047 (94.7)	2683 (90.8)	2106 (91.0)	7974 (93.4)	31935 (94.6)
**Hepatitis B vaccine, n(%)**					
Yes	3014 (11.9)	603 (20.4)	383 (16.6)	1309 (15.3)	4022 (11.9)
No	22390 (88.1)	2352 (79.6)	1931 (83.4)	7228 (84.7)	29745 (88.1)
**Pneumococcal vaccine, n(%)**					
Yes	8849 (34.8)	1207 (40.8)	1015 (43.9)	2618 (30.7)	11101 (32.9)
No	16555 (65.2)	1748 (59.2)	1299 (56.1)	5919 (69.3)	22666 (67.1)
**Influenza vaccine, n(%)**					
Yes	4924 (19.4)	505 (17.1)	560 (24.2)	1458 (17.1)	6184 (18.3)
No	20480 (80.6)	2450 (82.9)	1754 (75.8)	7079 (82.9)	27583 (81.7)
**TDaP (Tetanus, Diphtheria, and Pertussis), n(%)**					
Yes	8118 (32.0)	1094 (37.0)	854 (36.9)	2432 (28.5)	10425 (30.9)
No	17286 (68.0)	1861 (63.0)	1460 (63.1)	6105 (71.5)	23342 (69.1)
**Lack of immunization, n(%)**					
0	183 (0.7)	32 (1.1)	22 (1.0)	89 (1.0)	243 (0.7)
1	1074 (4.2)	214 (7.2)	150 (6.5)	439 (5.1)	1403 (4.2)
2	2753 (10.8)	366 (12.4)	344 (14.9)	771 (9.0)	3456 (10.2)
3	3954 (15.6)	470 (15.9)	415 (17.9)	1071 (12.5)	4924 (14.6)
4	4884 (19.2)	627 (21.2)	448 (19.4)	1724 (20.2)	6521 (19.3)
5	12556 (49.4)	1246 (42.2)	935 (40.4)	4443 (52.0)	17220 (51.0)
* The total number of CLD is calculated based on the unique patient ID. Those patients who reported multiple CLDs were counted only once.					

Table S7: Characteristics of all CLD cohort according to comorbidities

**Table S7:** Characteristics of all CLD cohort according to comorbidities					
	**NAFLD**	**ALD**	**Hepatitis B**	**Hepatitis C**	**CLD***
**Type 2 Diabetes, n(%)**					
No	13655 (54.4)	1610 (55.1)	1434 (62.5)	5830 (68.8)	19857 (59.4)
Yes	11443 (45.6)	1313 (44.9)	861 (37.5)	2642 (31.2)	13561 (40.6)
**Hypertension, n(%)**					
No	6923 (27.3)	603 (20.4)	695 (30.0)	3037 (35.6)	10337 (30.6)
Yes	18481 (72.7)	2352 (79.6)	1619 (70.0)	5500 (64.4)	23430 (69.4)
**BMI, n(%)**					
<30	9618 (40.2)	1595 (58.2)	1377 (61.6)	5295 (64.4)	14994 (46.9)
30+	14303 (59.8)	1146 (41.8)	860 (38.4)	2926 (35.6)	16970 (53.1)
**Sleep apnea, n(%)**					
No	15941 (62.7)	2223 (75.2)	1723 (74.5)	7075 (82.9)	23002 (68.1)
Yes	9463 (37.3)	732 (24.8)	591 (25.5)	1462 (17.1)	10765 (31.9)
**Hypothyroidism, n(%)**					
No	19067 (85.9)	2408 (90.8)	1888 (88.7)	7396 (93.2)	26419 (88.0)
Yes	3134 (14.1)	243 (9.2)	241 (11.3)	537 (6.8)	3599 (12.0)
**Comorbidities, n(%)**					
0	2574 (10.1)	344 (11.6)	413 (17.8)	2019 (23.6)	4835 (14.3)
1	5431 (21.4)	819 (27.7)	612 (26.4)	2589 (30.3)	8126 (24.1)
2	6380 (25.1)	831 (28.1)	609 (26.3)	2049 (24.0)	8321 (24.6)
3	6121 (24.1)	583 (19.7)	420 (18.2)	1219 (14.3)	7106 (21.0)
4	4220 (16.6)	334 (11.3)	218 (9.4)	582 (6.8)	4656 (13.8)
5	678 (2.7)	44 (1.5)	42 (1.8)	79 (0.9)	723 (2.1)
* The total number of CLD is calculated based on the unique patient ID. Those patients who reported multiple CLDs were counted only once.					

Figure S1

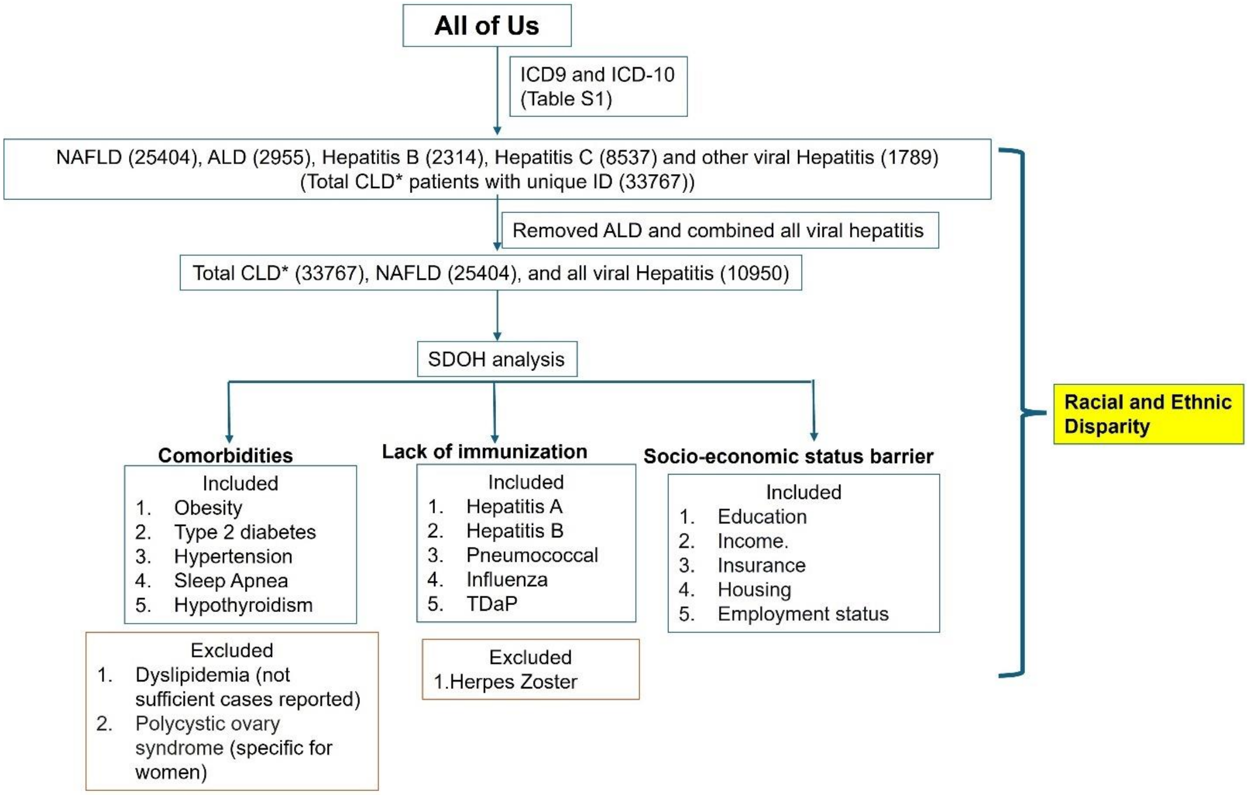

Figure S1. Flow chart of the All of Us data analysis.

*The total number of CLD is calculated based on the unique patient ID. Those patients who reported multiple CLDs were counted only once.

Figure S2

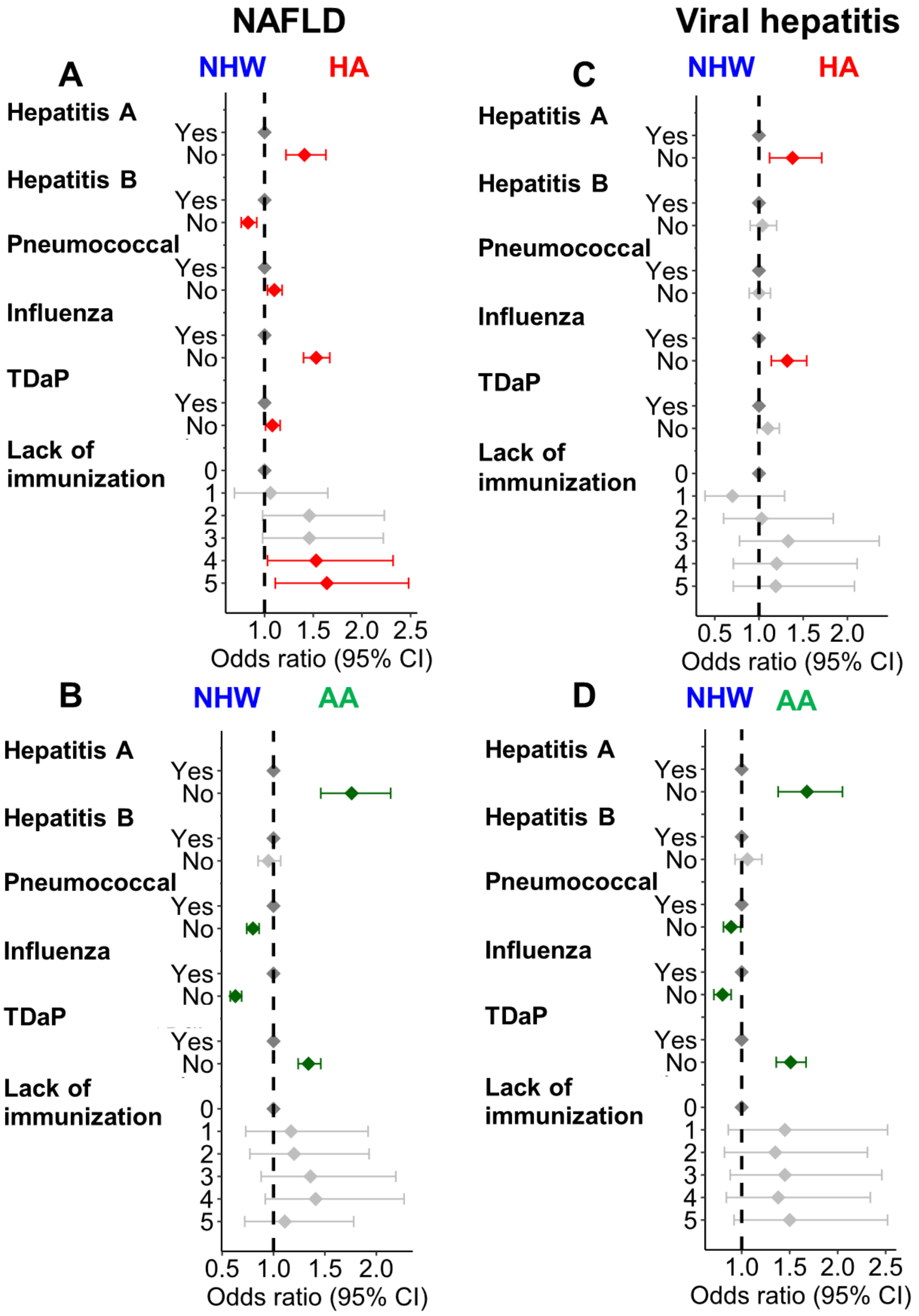

Figure S2. Age at diagnosis, gender, and insurance status adjusted Odds Ratio (OR) of lack of immunization in HA and AA CLD patients compared to NHW patients. A-B. Represents the OR of lack of immunization and NAFLD prevalence among HA (A) and AA (B). C-D. Represents the OR of lack of immunization and prevalence of viral hepatitis among HA (C) and AA (D). This analysis was not performed in the AN population due to <20 participants in some of the categories. The red and green colors represent significant OR ($p<0.05$) in HA and AA, respectively, compared to NHW.

Figure S3

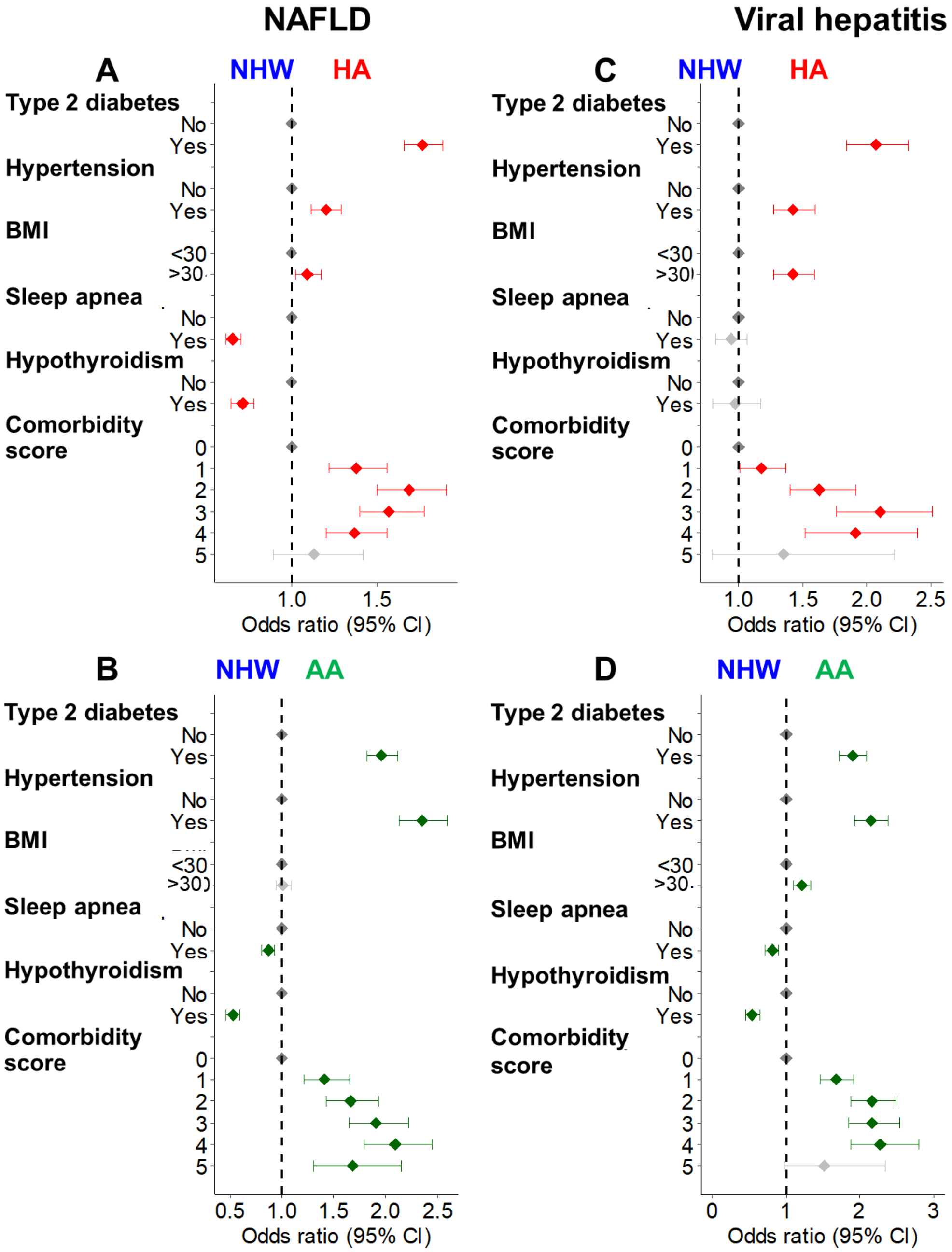

Figure S3. Age at diagnosis and gender-adjusted OR of comorbidities in HA and AA patients compared to NHW patients. A-B. Represents the association of NAFLD prevalence among HA (A) and AA (B) and various comorbidities. C-D. The ORs regarding comorbidity score for HA (C) and AA (D) among viral hepatitis patients. E-F. The comorbidity association in viral Hepatitis HA (E) and AA (F) patients. This analysis was not performed in the AN population due to <20 participants in some categories. The red and green colors represent significant OR ($p<0.05$) in HA and AA, respectively, compared to NHW.

Figure S4

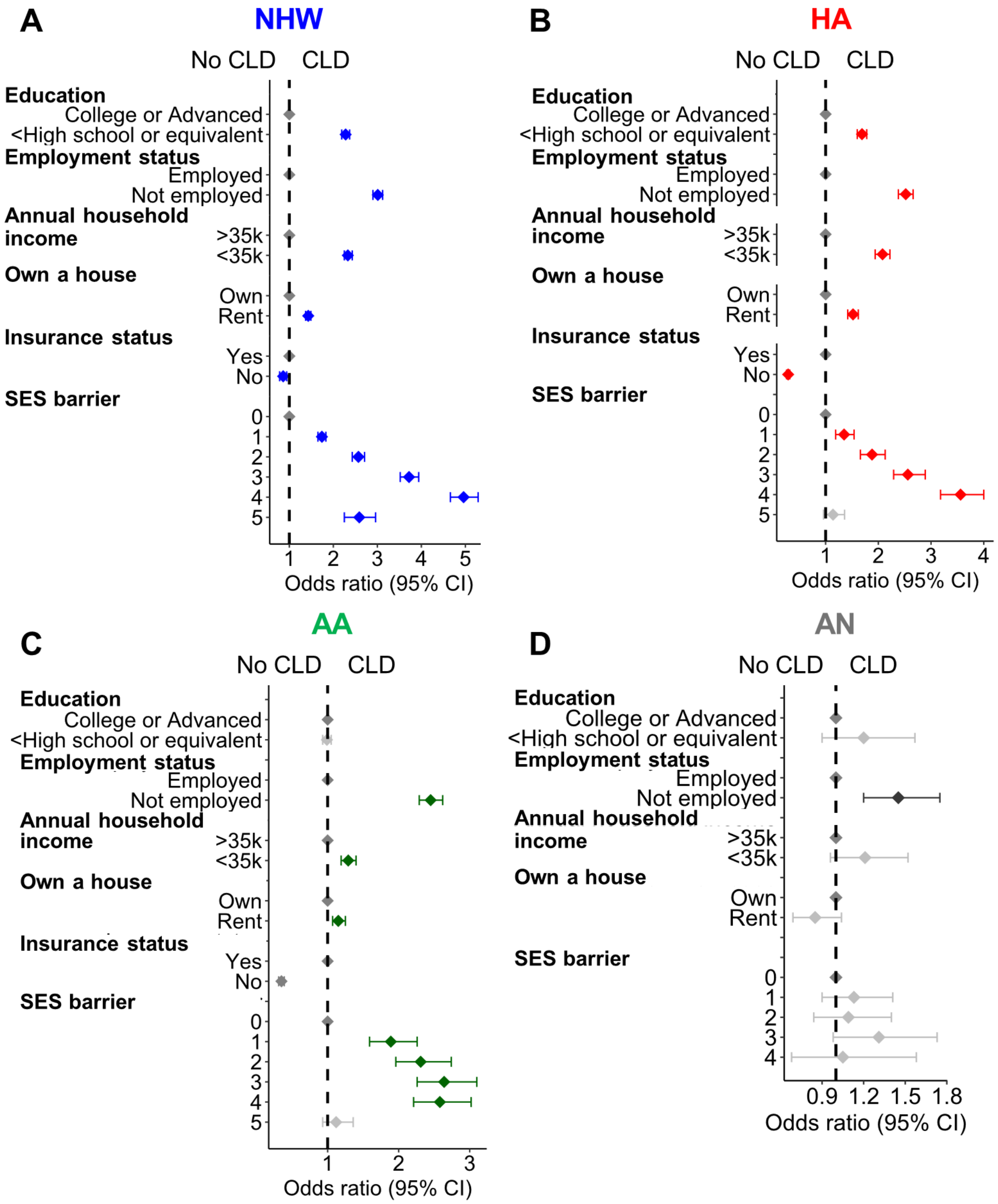

Figure S4. Age at diagnosis and gender-adjusted OR of SES barriers in HA (A), AA (B), AN (C), and NHW (C) CLD patients compared to their age, gender and race-matched non-CLD controls without any obesity (BMI >30), Type 2 diabetes, hypertension, sleep apnea, and hypothyroidism (comorbidity score '0').

Figure S5

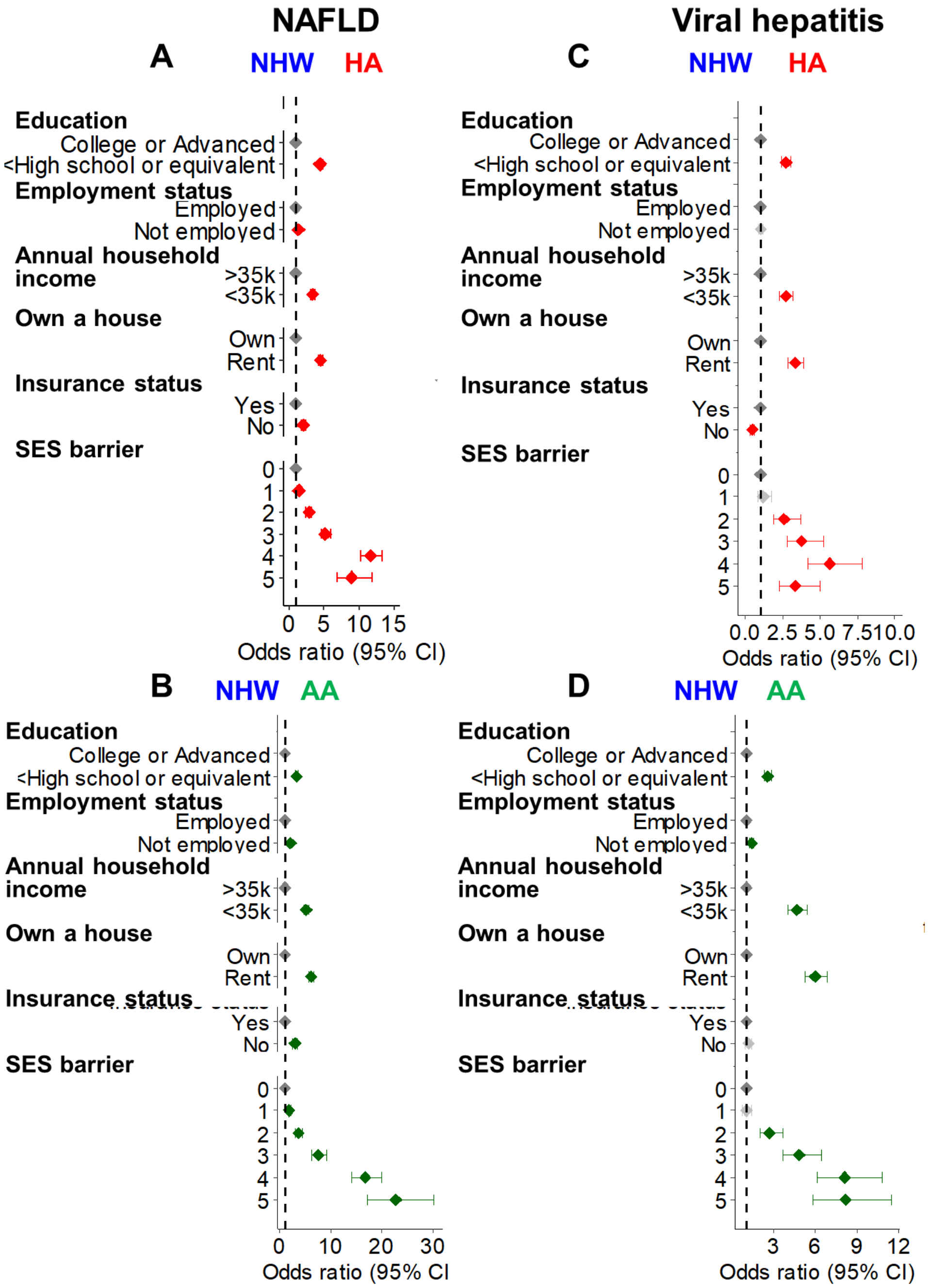

Figure S5. Age at diagnosis and gender-adjusted OR of SES barriers in HA and AA patients compared to NHW. A-B. The association of SES barriers and NAFLD prevalence among HA (A) and AA (B) are plotted here. C-D. The ORs for SES barriers in viral hepatitis HA (C) and AA (D).This analysis was not performed in AN population due to <20 participants in some categories. The red and green colors represent significant OR ($p<0.05$) in HA and AA, respectively, compared to NHW.

Figure S6

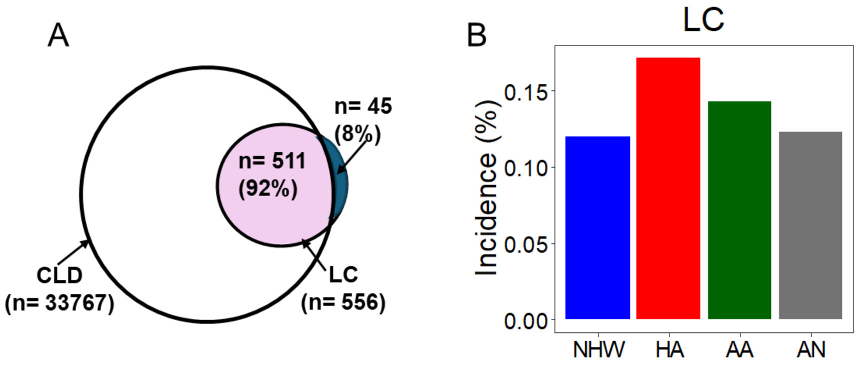

Figure S6. A. The overlap of CLD and LC patients in ‘All of Us’ data. B. Racial distribution of ‘All of Us’ participants reported LC.

Figure S7

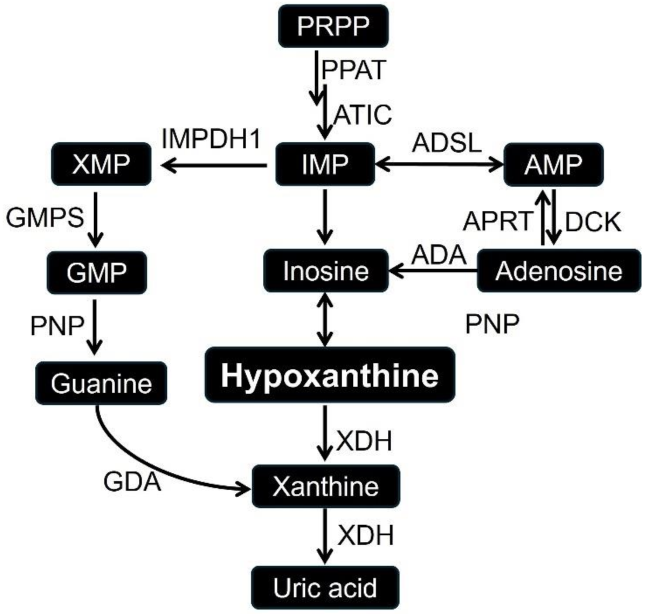

Figure S7. Purine Metabolism Pathway metabolites and enzymes.

PRPP (Phosphoribosyl Pyrophosphate), XMP (Xanthosine monophosphate), IMP (Inosine monophosphate), GMP (Guanosine monophosphate), AMP (Adenosine monophosphate), PPAT (Phosphoribosyl Pyrophosphate Amidotransferase), ATIC (5-Aminoimidazole-4-Carboxamide Ribonucleotide Formyltransferase/IMP Cyclohydrolase), IMPDH1 (Inosine Monophosphate Dehydrogenase 1), ADSL (Adenylosuccinate Lyase), GMPS (Guanine Monophosphate Synthase), APRT (Adenine Phosphoribosyltransferase), DCK (Deoxycytidine Kinase), PNP (Purine Nucleoside Phosphorylase), GDA (Guanine Deaminase), and XDH (Xanthine Dehydrogenase).

Figure S8

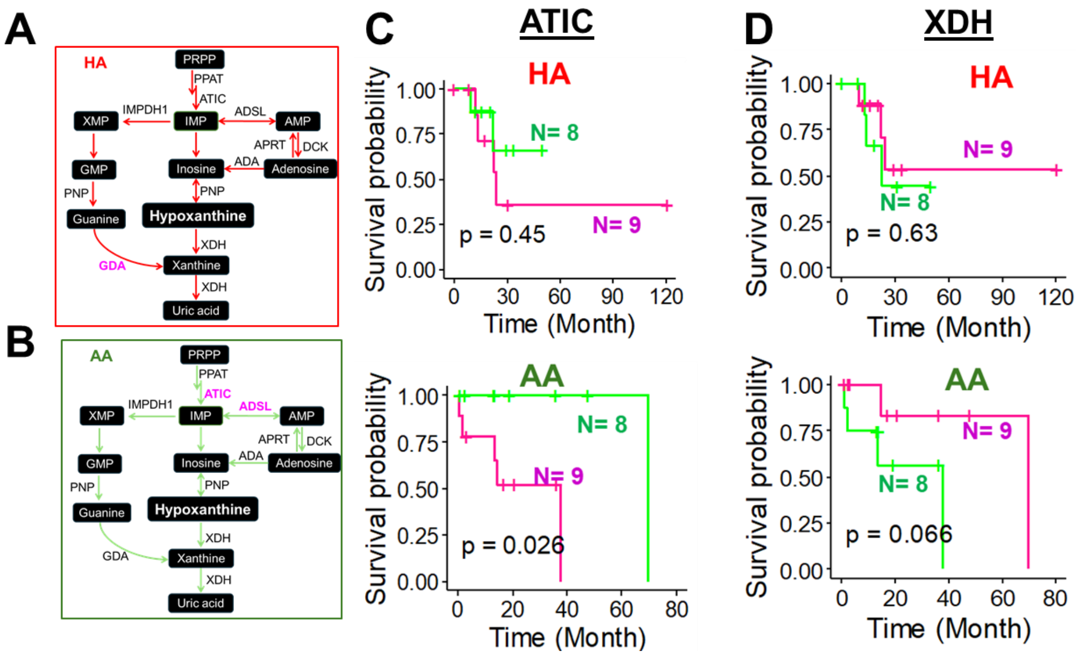

Figure S8: A-B. The enzymes involved in the purine metabolism pathway were analyzed in the TCGA data among HA (A) and AA (B) LC patients. The pink color letters represent the enzymes associated with poor survival at higher mRNA expression. The black color letters represents enzymes with no prognostic significance. C-D. Kaplan-Meier survival curve of OS of ATIC (C) and XDH (D) mRNA in HA and AA LC patients. Median mRNA expression separated high (pink) and low (green) sub-groups.

Figure S9

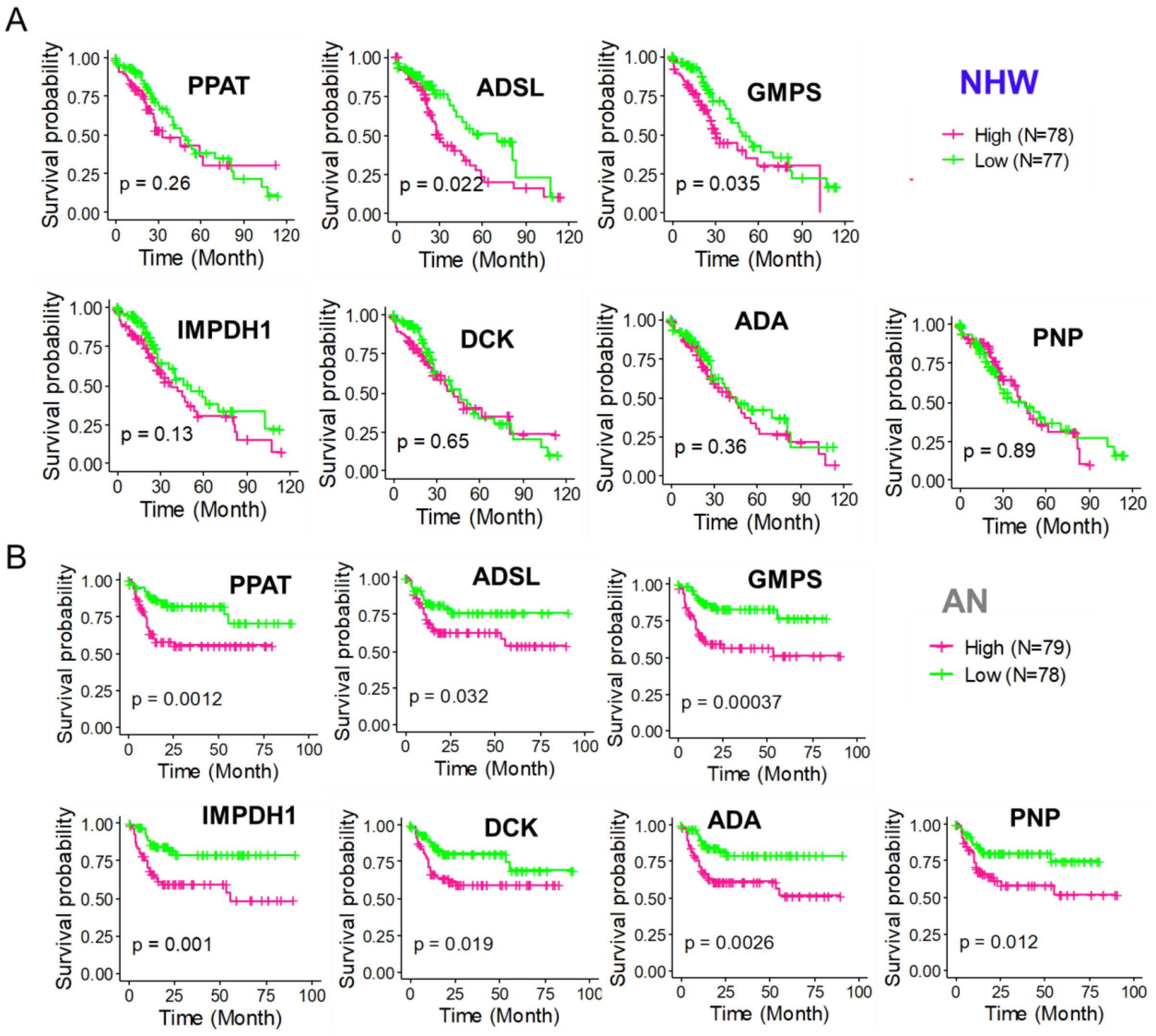

Figure S9: Kaplan-Meier survival curve of OS of purine metabolism genes in NHW (A) and in AN (B) LC patients. Median mRNA expression separated high (pink) and low (green) sub-groups.

Figure S10

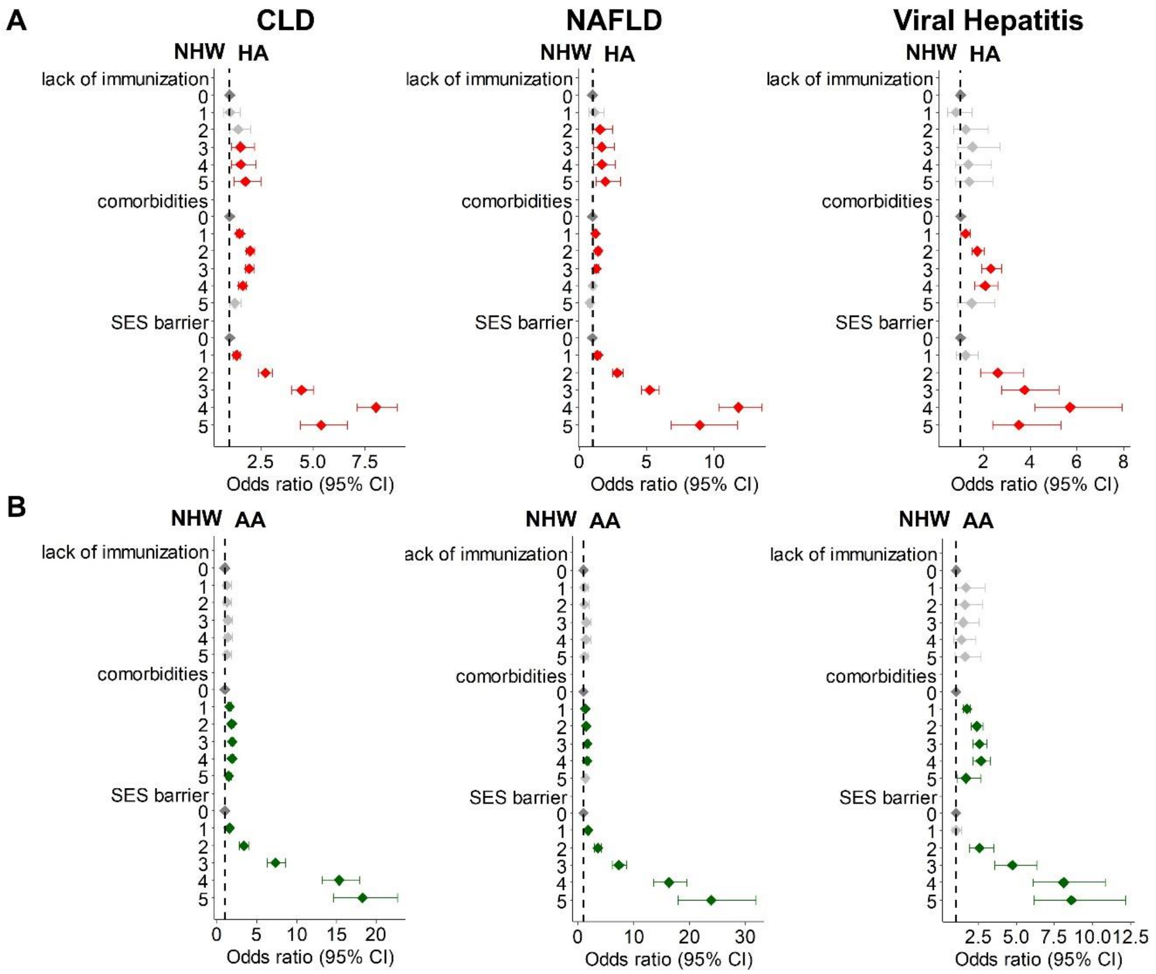

Figure S10: The OR of the three confounding factors (lack of immunization, comorbidities, and SES barrier) among (A) HA and (B) AA CLD, NAFLD, and viral hepatitis patients compared to NHW. Each confounding factor was adjusted to Age at diagnosis, gender, and the other two factors.
